# A Case of Lobar Pneumonia and Its Treatment

**Published:** 1899-05-20

**Authors:** G. A. Sutherland

**Affiliations:** Physician to Paddington Green Children's Hospital


### Hospital Clinics and Medical Progress.

### A CASE OF LOBAR PNEUMONIA AND ITS TREATMENT.

By G. A. Sutherland, M.D., M.R.C.P., Physician to Paddington Green Children's Hospital.

(*Continued from page* 110.)

Pneumonia is now regarded as a general infective disorder with local manifestations in the lungs, just as typhoid fever is a general disease with local lesions in the bowel. We do not adopt active local treatment for typhoid ulceration, or for pneumonic consolidation. Rest in bed, careful dieting, protection from cold, and soothing remedies for pain are the lines of treatment under which the majority of cases of lobar pneumonia in childhood make a speedy and complete recovery.

As is common in adults, one lobe of the lung only may be affected; but sometimes in children we find that the disease has a tendency to spread from lobe to lobe until the whole of one lung is involved, or from lung to lung until both lungs have been affected in their entirety, as in this case. One point is to be noted in this connection—namely, that from the time of the onset of the acute illness until the whole of the right lung had been involved there was no cessation of the continuous pyrexia or the general prostration, whereas in the later attacks involving first the left upper lobe and then the left lower lobe the temperature was not so high, and prostration was not so marked a feature. These later attacks appeared to be due to local inflammation without much constitutional disturbance. The occurrence of spreading pneumonia has been a marked feature in connection with epidemics of influenza, and possibly influenza may have been a factor in this case. But, as I said, spreading pneumonia involving lobe after lobe is by no means rare in children, whereas in influenza the consolidation is usually of a more patchy nature involving frequently both lungs at the same time.

As regards the complications present in this case, at an early stage of the illness both middle ears were found to be distended with fluid, one of them containing pus. This infection of the ear from the throat is a common complication of lobar pneumonia in children, so much so that I always ask my colleague, Dr. Lambert Lack, to examine the ears of all patients who may be admitted suffering from that disorder. This is not done only when they present symptoms of ear trouble, such as pain in the head or ear, rolling of the head, &c., because in a great number of cases we find that those symptoms are entirely absent, and yet on examination the membranes may be found inflamed and bulging from the presence of pent-up fluid. In pneumonia there may exist acute pneumococcic inflammation of the middle ear which leads to no complaint on the part of the patient, which does not attract notice by any symptoms, and which is only discovered by objective examination. This ought to be carried out as a matter of routine in all cases.

Again, in connection with pneumonia of adult life, when acute delirium supervenes, the possibility of an alcoholic basis at once suggests itself. In a similar way, when in the course of lobar pneumonia in children, delirium appears with signs of meningeal irritation such as restlessness, slight twitching of the facial or finger muscles, drowsiness with irritability, &c., those symptoms are frequently dependent on acute inflammation of the middle ear. If we make a diagnosis at once of meningitis and treat that condition only we run the risk of overlooking the cause of the symptoms, and one which is much more amenable to treatment. We have had a number of cases in which puncture of the tympanic membranes has led speedily to the disappearance of all cerebral symptoms and has probably, in some of the cases, prevented the spread of the inflammation to the meninges.

At the necropsy in this case pus was found to be present in three different organs—an empyema around the lower lobe of the left lung, purulent meningitis enveloping most of the surface of the brain, and suppuration in the left middle ear and adjacent mastoid cells. The empyema was secondary to the pneumonia of the adjoining lobe; the purulent otitis was due to infection spreading from the naso-pharynx. What was the cause of the purulent meningitis? In some cases of empyema secondary involvement of the brain occurs, usually in the form of a cerebral abscess, but such an occurence is very rare. On the other hand, the extension of inflammation, whether simple or purulent, of the middle ear to the meninges and brain is but too common. In the early part of this patient's illness, as you will remember, both ears were opened by slitting the tympanic membranes, and the discharge had continued for some days. In the case of the right ear the discharge was purulent, and at the necropsy this ear was found to be apparently healthy. On the other hand, in the case of the left ear there had been no purulent discharge, the membrane had healed, there were no signs of ear trouble later on to direct our attention to that organ, and yet at the necropsy it was found to be full of pus. We were concentrating our attention on the pulmonary condition, but had we continued our observation of the ears we might have been able to evacuate the pus externally, and prevent its extension internally with consequent purulent meningitis. This is a further instance of the insidiousness of ear mischief in connection with pneumonia, and enforces the necessity not only of an early examination of the ears, but of repeated examination when the illness is prolonged.

